# Pericardial cyst: an unusual cause of pneumonia

**DOI:** 10.1186/1757-1626-1-26

**Published:** 2008-07-10

**Authors:** Wael Faroug Elamin, Kieran Hannan

**Affiliations:** 1 Department of Medicine, Cavan General Hospital, Cavan, Ireland

## Abstract

**Introduction:**

Pericardial cysts occur rarely with an incidence rate of 1 per 100,000. They are usually detected by chance and are clinically silent in most cases.

**Case presentation:**

We report the case of a 66 year old Irish male, with signs and symptoms of pneumonia. A chest X-Ray confirmed left lower lobe consolidation. Further imaging including a CT thorax identified a large pericardial cyst as the cause of the symptoms.

**Conclusion:**

Although pericardial cysts are usually asymptomatic, they can present with chest pain and symptoms of respiratory tract infection.

## Background

Pericardial cysts are uncommon benign congenital anomalies. They are the most common benign tumours of the pericardium. They are usually identified on the third or fourth decade of life and are equally common in males and females. Clinically and radiologically they resemble other tumours of the pericardium. CT scan, Echocardiography and MRI are the most useful investigations.

## Case presentation

A 66 year old Irish male, non-smoker, presented to the emergency department complaining of left sided pleuritic chest pain, associated with non productive cough for two days. His past medical history included hypertension, hypercholesterolemia, varicose veins, and migraine. Five years previously he was admitted for "chest pain" and all of his investigations including coronary angiography were normal. Seven months prior to this presentation he suffered an anaphylactic reaction to a Wasp sting. His chest X-Ray was normal. He had significant family history of heart disease as both parents suffered from myocardial infarction.

**Figure 1:**
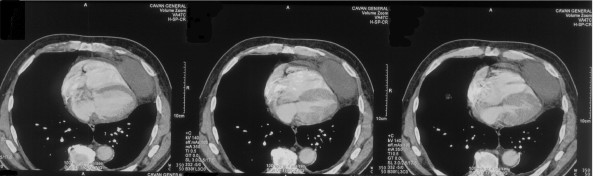
CT Scan of the thorax showing the pericardial cyst.

**Figure 2:**
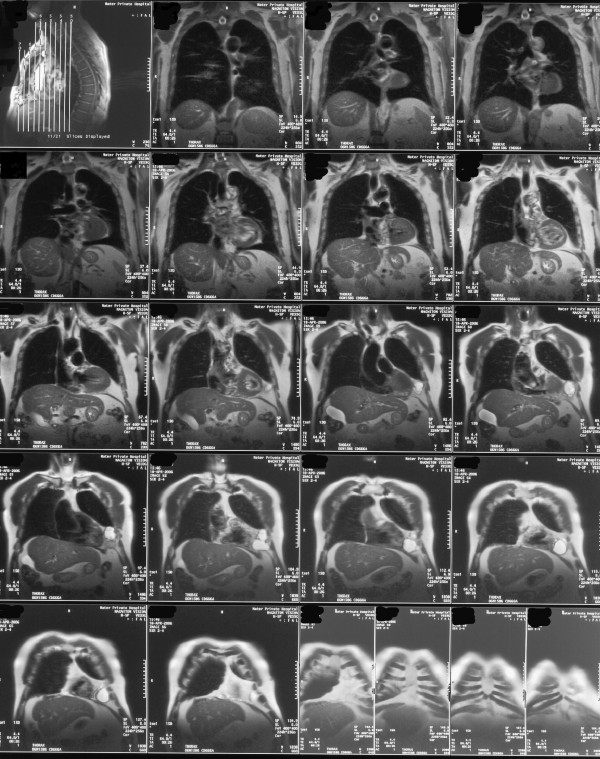
MRI Scan of the thorax showing the pericardial cyst.

His ECG revealed T wave flattening in leads III and AVF (old changes), chest x-ray showed left lower lobe consolidation. Routine bloods including full blood count, urea and electrolytes, and liver function tests were all within normal range. Arterial blood gas was normal. CRP was elevated at 79.9 mg/L. The patient was commenced on intravenous antibiotics and a CT thorax was requested. It showed an elliptical well defined shadow (figure 1), with mixed internal density lying above the diaphragm and closely related to the left cardiac margin, most likely representing a pericardial cyst. An MRI of the thorax (figure 2) showed the cyst to be 7.9 cm × 3.6 cm × 6.0 cm. The patient remained symptomatic and was therefore referred to the cardiothoracic team for excision of the cyst, which was performed successfully.

## Discussion

Pericardial cysts occur at the rate of 1 person per 100,000 [1]. They result from failure of fusion of one of the mesenchymal lacunae that form the pericardial sac. Seventy five percent have no associated symptoms, and are usually found incidentally during routine chest x-ray or echocardiography. Seventy percent are located at the right costophrenic angle, 22% on the left and the remaining in the posterior or anterior superior mediastinum [2]. It is unknown if the size or position of the cyst corresponds to higher rates of complications. Pericardial cysts are usually asymptomatic, and are usually found incidentally on routine chest x-rays. Episodes of chest pain, tachycardia, persistent cough, cardiac arrhythmias and lower respiratory tract infection have been described. The symptoms can result from the pressure of the cyst on the adjacent organs [3]. Other reported complications include right ventricular outflow obstruction, inflammation and infection, pulmonary stenosis, partial erosion into adjacent structures, atrial fibrillation, and congestive heart failure [4-7]. Some pericardial cysts resolve spontaneously, most likely from rupture into the pleural space. The rates of spontaneous resolution, growth or complication have not been reported. In our patient a pericardial cyst of 7.9 cm × 3.6 cm × 6.0 cm appeared within a 7 month period and became symptomatic.

Contrast CT scan is the modality of choice for diagnosis and follow-up [1,4]. However, no studies have been performed to confirm the superiority of contrast CT over MRI or echocardiography for diagnosis and follow-up. On CT scan, pericardial cysts appear as thin-walled, sharply defined, oval homogeneous masses. They fail to enhance with intravenous contrast [3].

The management of pericardial cysts includes observation, per-cutaneous drainage, and resection. The indications of resection include large size, symptoms, patient concern, uncertainty of malignant potential and prevention of life threatening emergencies such as cardiac tamponade, obstruction of right main stem bronchus and sudden death. Thoracoscopy is the approach of choice [8].

## Conclusion

While pericardial cysts are rarely the cause of respiratory tract signs and symptoms never the less this case highlights the importance of further imaging especially on the background of an abnormal chest X-Ray. CT scans are useful in confirming the diagnosis. Definitive management for symptomatic cysts is surgical resection.

## Consent

"Written informed consent was obtained from the patient for publication of this case report and accompanying images. A copy of the written consent is available for review by the Editor-in-Chief of this journal".

## Competing interests

The authors declare that they have no competing interests.

## Contributions by Authors

WE prepared the manuscript. KH was involved with the patient management, reviewed the manuscript and made the final corrections before submission.
